# Use of smartphone-based instant messaging services in medical practice: a cross-sectional study

**DOI:** 10.1590/1516-3180.2020.0228.15052020

**Published:** 2020-06-22

**Authors:** Thiago Gonçalves dos Santos Martins

**Affiliations:** I MD. Physician and Doctoral Student, Department of Ophthalmology, Universidade Federal de São Paulo (USP), São Paulo (SP), Brazil; Doctoral Student, Universidade de Coimbra (UC), Coimbra, Portugal.

Dear Editor,

In response to the article titled “Use of smartphone-based instant messaging services in medical practice: a cross-sectional study” published in your esteemed journal, which is a well thought-out and well-written paper, I would like to raise few points regarding this study.

The article reported that doctors and medical students have used instant messaging services to discuss clinical cases, allow interactions between healthcare professionals and patients or disseminate knowledge. However, ethical and legal limitations to the use of telemedicine need to be discussed.^1^

Telemedicine can be used to serve the population and can be used by healthcare professionals in the field of ​​education. Advances in medical legislation will be necessary for enabling national certification of doctors so that they can work both nationwide and internationally.

Moreover, security in electronic systems needs to be guaranteed so as to preserve patient data. Unfortunately, encrypted information systems remain much less secure than those based on old paper records.

Care is needed to ensure that telemedicine is not used just to reduce investment in healthcare but is used to increase the efficiency of the provision of medical services. The list of activities that can be conducted via telemedicine and the mandatory technical requirements for these activities need to be widely discussed. These requirements need to be adhered to, so as to ensure that work done through telemedicine is of good quality. Verbal consent needs to be obtained, so that the scope of what telehealth entails is delineated, along with the benefits and risks of using telehealth to perform limited examinations, and any other state-designated requirements. Situations in which these examinations are not live and are therefore incomplete should be highlighted.

In this manner, an excellent return can be obtained for society, thereby ensuring continuous education for healthcare professionals and facilitating healthcare management. Telemedicine can be useful for saving lives through social distancing in a hyperendemic area, while also remaining available to provide care for needy patients.^2^

However, doctors who use telemedicine are responsible for the quality of patient care and should not opt ​​for telemedicine consultations unless they consider that this is the best option available. To make this decision, they should take into account quality, access and cost. This new technology brings a variety of benefits, including: acquisition of new knowledge by the healthcare team, diagnoses for patients in regions with a shortage of specialists and avoidance of long-distance travel for doctors and patients.
